# Chilaiditi syndrome

**DOI:** 10.11604/pamj.2017.26.129.11557

**Published:** 2017-03-07

**Authors:** Hicham Naji-amrani, Aziz Ouarssani

**Affiliations:** 1 Department of Pneumology, Moulay Ismail Military Hospital, Meknes, Morocco

**Keywords:** Chilaiditi, colon, subphrenic interposition

## Image in medicine

Chilaiditi syndrome or subphrenic interposition of the colon, is a rare condition with an incidence of 0.025% - 0.28% in radiographs and mostly diagnosed as an incidental finding. We report the case of a 67-years-old male, heavy smoker, with history of intermittent abdominal pain, who reported cough and muco-purulent sputum without fever or dyspnea. Physical examination showed bilateral bronchial rales and right hypochondrium sensitivity, his vital signs were normal. A posteroanterior chest films (A) reveled an elevated right hemidiaphragm with free gas below. A pneumoperitoneum, subphrenic abscess, and intestinal pneumatosis were suspected, however, a CT scan of the thorax and abdomen (B, C and D) showed the interposition of the right colon angle between the diaphragm and the liver without any perforated viscus. Our patient was treated for chronic bronchitis infection; otherwise his Chilaiditi syndrome required symotomatic treatment with good outcomes.

**Figure 1:**
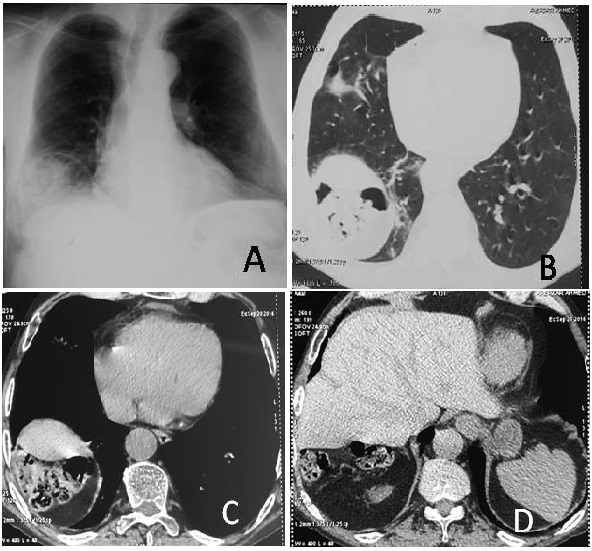
(A) posteroanterior chest films shows gas under elevated right hemidiaphragm; (B; C and D0; CT scan showing the interposition of right colic angle above the liver dome

